# The 2022 AMN Virtual Congress – Day 2

**DOI:** 10.25122/jml-2022-1010

**Published:** 2022-07

**Authors:** Stefana-Andrada Dobran, Alexandra-Mihaela Gherman, Dafin Fior Muresanu

**Affiliations:** 1. RoNeuro Institute for Neurological Research and Diagnostic, Cluj-Napoca, Romania; 2. Sociology Department, Babes-Bolyai University, Cluj-Napoca, Romania; 3. Department of Neuroscience, Iuliu Hatieganu University of Medicine and Pharmacy, Cluj-Napoca, Romania

## INTRODUCTION

The second day of the 2022 Academy of Multidisciplinary Neurotraumatology (AMN) Virtual Congress began with Session 4, with Prof. Karin Diserens (Switzerland) and Dr. Pieter Vos (the Netherlands) as chairpersons. The day encompassed a plethora of presentations on TBI, with the speakers approaching subjects such as disorders of consciousness, the role of Brain-Derived Neurotrophic Factor (BDNF), diffuse axonal injury, the impact of TBI on the Alzheimer's Disease, personalized approaches to TBI management, traumatic brain hemorrhage, the role of oxiracetam nanodelivery for the enhancement of memory, the complications of decompressive craniotomies following TBI, neurovascular therapies and the innovative PRESENT (Patient REgistry – Short Essential NeuroTrauma). Moreover, the two Panels of the second day brought together respected specialists who discussed the focus of the AMN as a society and on TBI in low- and-middle-income countries.

## CONGRESS SESSIONS

### Session 4

The first presentation regarded the modern concepts of disorders of consciousness, where Karin Diserens (Switzerland), Head of the Acute Neuro-rehabilitation Unit at the Department of Clinical Neurosciences from Lausanne University Hospital (Switzerland), shared her expertise on neurotrauma. The pathologic alteration in consciousness represents one of the most challenging aspects of TBI since the simple definition of consciousness during history proved to be problematic from a scientific point of view and from a philosophical one. The evolution of medicine has led to better outcomes secondary to brain injuries, prolonging survivability. Several aspects of the disorders of consciousness have led to a better and deeper understanding of the Disorders of Consciousness (DOC) from an anatomical, biological, and functional perspective.

In modern medicine, the concepts of DOC have changed with the help of electroencephalogram (EEG) or functional Magnetic Resonance Imaging (MRI) studies and translational neurology. These concepts are a mirror to the continuous development of medicine.

Secondly, Anton Álvarez (Spain), Director at Medinova Institute of Neurosciences and Clinical Research and Director at QPS-JSW Life Sciences (Spain), presented an intriguing insight into the role of Brain-Derived Neurotrophic Factor (BDNF) in clinical and basic research. BDNF represents the most abundant neurotrophin in the brain with an essential role in synaptic plasticity, cell differentiation and survival, playing a vital role in neuropathology and treatment and exerting a positive influence on TBI neurorecovery by promoting endogenous processes of regeneration, thus enhancing survival, and improving TBI outcomes. It is, therefore, essential to enhance BDNF in the brains of TBI patients to improve cognition and prevent dementia after TBI. Lastly, it is crucial to consider the shift from acute neuroprotection to sub-acute and chronic neurorestoration.

Moreover, Pieter Vos (the Netherlands), Neurologist at the Department of Neurology at Slingeland Hospital in Doetinchem (the Netherlands), conducted a fascinating presentation regarding diffuse axonal injury. Diffuse axonal injury is one of the significant complications of TBI that can lead to impairment and death. With an unknown incidence, this insult to the brain leads to malfunction of the neuronal networks, affecting various functional structures of the brain. Diffuse axonal injury is found mainly in severe TBI cases, predominantly affecting the white matter of the frontal and temporal lobes and the structures of the brainstem and corpus callosum. The definitive diagnosis is made post-mortem, although imagery like Computed Tomography (CT) or MRI can identify various lesions secondary to diffuse axonal injury. Therefore, Dr. Vos highlighted that for TBI patients with diffuse axonal injuries, preventing secondary injuries and providing physical, neuropsychological, and pharmacological rehabilitation is of utmost importance in decreasing morbidity and mortality.

The session ended with a presentation by Alexandru V. Ciurea (Romania), Vice President of the Romanian Ministry of Health Neurosurgical Committee, Chief of Neurosurgery and Scientific Director at Sanador Medical Center Hospital in Bucharest and Professor of Neurosurgery at Carol Davila University in Bucharest (Romania), on the role of TBI as a trigger or aggravating factor in Alzheimer's disease. Several pathophysiological aspects of TBI remain an enigma. Thus, the gap between translational and clinical research represents a need, an opportunity, and the future of TBI. A close collaboration between clinicians and translational researchers will lead to the further development of this field, mainly by finding comparable data between human and animal models. Prof. Ciurea presented a current study on Alzheimer's Disease and TBI on 19 patients, from 2013 to 2022, showcasing that TBI is an aggravating factor for Alzheimer's disease, as it affects synaptic transmission. Prof. Ciurea discussed further prevention strategies for Alzheimer's Disease.

### Session 5

During the 5^th^ session, Prof. Christian Matula (Austria) and Prof. Martin Rakusa (Slovenia) represented the chairpersons, and exciting topics related to neurosurgical aspects of TBI and inspiring approaches towards TBI were showcased.

At the beginning of the session, Felix Brehar (Romania), Head of the Stereotactic and Functional Neurosurgery Department at Bagdasar Arseni Clinical Emergency Hospital in Bucharest and Associated Professor at Carol Davila University of Medicine and Pharmacy in Bucharest (Romania), presented current and future trends toward a personalized approach to TBI management. A successful individualized approach should pinpoint the presence, the severity, the possibility that the patients would recover on their own, the best treatment option, and what the long-term disability would be. A personalized approach integrates clinical diagnosis, multi-omics approach, and tailored treatment. The multi-omics integrates data on genomics, epigenomics, transcriptomics, proteomics, and metabolomics. Prof. Brehar showcased the role of biomarkers in TBI in the diagnosis, stratification, delivery of targeted therapy, and prognosis. A take-home conclusion from the presentation regards the variability in TBI, the need for further research and the importance of multimodality and interdisciplinary efforts in the approach of TBI.

Bringing forth another inspiring approach, Martin Rakusa (Slovenia), Consultant Neurologist in the Division of Neurology and Head of the Medical Research Department at University Medical Centre Maribor (Slovenia) discussed the topic of traumatic brain hemorrhage. Prof. Rakusa pinpointed the rarity of traumatic hemorrhage in TBI, with a more common occurrence in more severely affected patients, as well as the lack of association of anticoagulants with hemorrhage. An equally insightful presentation was that of Hari Shanker Sharma (Sweden), director of Research at the International Experimental Central Nervous System Injury & Repair (IECNSIR) at Uppsala University. Prof. Sharma discussed on the role of oxiracetam nanodelivery for the enhancement of memory, functional recovery, and induction of neuroprotection following a concussive head injury. The 5^th^ session of the Congress ended with the skillful presentation of Prof. Stefan Florian (Romania), Head of the Department of Neurosurgery at Cluj County Emergency Hospital and Vice President of the Romanian Society of Neurosurgery. Prof. Florian delivered a detailed perspective regarding the complications of decompressive craniectomy in TBI.

### Session 6

The last session of the AMN Congress, Session 6, having Prof. Michael Chopp (USA) and Prof. Hari Shanker Sharma (Sweden) as chairpersons, discussed neurovascular therapies and the innovative TBI registry, PRESENT. The first speaker of the session, Michael Chopp (USA), Vice Chairman for Research of the Department of Neurology and Scientific Director of the Henry Ford Neuroscience Institute in the USA, discussed the topic of exosomes and neurotrophic factors in Neurovascular Therapy. On a microscopic level, exosomes represent a valuable structure with various functions. For example, they can assure intercellular connection by transporting different nucleic acids, proteins, or lipids. Prof. Chopp further discussed on the progression of diseases, and potential biological markers of different lesions.

It was dr. Iulia Vadan (Romania), Neurologist at RoNeuro Institute for Neurological Research and Diagnostic in Cluj-Napoca (Romania), who then discussed the status of PRESENT. PRESENT, short for Patient Registry, Short Essential Neurotrauma, has been developed as an international, multidisciplinary longitudinal, prospective registry. The registry represents an electronic platform for TBI patients, covering the patient's pathway during the hospital stay and discharge, with possibility of implementation at national and international levels. PRESENT will be able to mirror the multimodal treatment in TBI. One of the main characteristics of TBI is the heterogeneity of mechanisms, causes, and types of brain injuries, as well as the patient's particularities that lead in a first phase to the primary injury and then to secondary injuries. Furthermore, the patient's rehabilitation after TBI is of utter importance by influencing the degree of posttraumatic disability and the patients' quality of life, as well as their families. Based on an electronic platform, it is a valuable tool in obtaining a TBI patients database that will cover several elements from epidemiology to the pathway of care, outcomes, treatments, and rehabilitation measures.

Finally, Prof. Andriy Huk (Ukraine), Chief of the Department of Endoscopic and Craniofacial Neurosurgery at Romodanov Neurosurgery Institute, painted an inspiring portrait of the role of PRESENT in the multimodal treatment following a TBI.

## PANELS

### 3^rd^ Panel

During the 3^rd^ Panel “The AMN is an ambitious medical society-what should be its focus?”, Volker Hömberg (Germany), Jongmin Lee (South Korea), Johannes Vester (Germany), Lynne Lucena (The Philippines), Christian Matula (Austria), Nicole von Steinbüchel (Germany), Pieter Vos (The Netherlands) and Dafin Muresanu (Romania) discussed the focus of AMN as a modern medical society. The AMN aims to advance research, practical application, and education in neurotraumatology and is determined to become a leading example of multidisciplinary cooperation. However, considering the complexity of TBI, especially with the fast-growing advancement of research and innovation, finding the right path for scientific societies can be a challenging pursuit.

The AMN Congress was finalized with the last of the panels, respectively Panel 4 “TBI in low- and middle-income countries – what are the specifics, what are the pragmatic solutions for improvements”, where panellists like Felix Brehar (Romania), Volodymyr Golyk (Ukraine), Ignacio Previgliano (Argentina) and Tarek Lotfy (Egypt) alongside members of the AMN board and specialists from Romania, including Andriy Huk (Ukraine), Johannes Vester (Germany), Nicole von Steinbüchel (Germany), Stefan Strilciuc (Romania), Razvan Chereches (Romania), Irina Vlad (Romania), Iulia Vadan (Romania), Constantin Radu (Romania) showcased the specific problems of neurotrauma in low- and middle-income countries (LMIC), as well as discussed possible approaches.

LMIC are more affected by TBI than more resourceful ones and experience specific challenges in practice and research, the availability and reliability of data. Pragmatic solutions, such as PRESENT are needed to obtain more profound knowledge about TBI.

Moreover, it is crucial to consider the needs and particularities of the specific countries when discussing TBI research and consider the patient's pathway through the medical system. Therefore, having an “ecological” approach and considering the larger picture when approaching TBI remains of utmost importance.

### Lessons from the AMN Congress 2022

The exciting event showcased insight into a wide-range of TBI-related themes, offering a glimpse into the complex and intricate world of neurotraumatology. The multidisciplinary principle of the AMN is reflected in the variance of perspectives presented, standing as the cornerstone of future research and medical practice. In a fast-changing and unpredicted environment, collaboration and dissemination of knowledge, skills, and innovation support the pursuit of science and bring optimistic perspectives for the future. More information on the congress can be found on the AMN website.

